# Spontaneous hypoglycemia: should we mind the gap? Long-term follow-up of healthy people who met Whipple’s triad criteria

**DOI:** 10.1007/s42000-024-00542-1

**Published:** 2024-03-08

**Authors:** Jan Adelmeyer, Christian Marcel Schauer, Peter Herbert Kann

**Affiliations:** 1 Center for Endocrinology, Diabetology & Osteology of Philipps, University Marburg, 35037 Marburg, Germany; 2 German Center for Endocrine Care (DEVZ), Düsseldorfer Str. 1-7, 60329 Frankfurt am Main, Germany

**Keywords:** 72-hour fast, Insulinoma, Spontaneous hypoglycemia, Follow-up

## Abstract

**Context:**

Patients discharged as “healthy” with the symptoms of spontaneous hypoglycemia, commonly known as Whipple’s triad, need more attention.

**Objective:**

Characterization and long-term follow-up of symptom development in patients with spontaneous hypoglycemia discharged as “healthy”. The objective was to ascertain whether any conditions related to the symptoms were diagnosed during the follow-up period.

**Methods:**

Retrospective analysis of patient data and evaluation of a specific questionnaire on the development of symptoms of spontaneous hypoglycemia. In addition, patient questionnaires were evaluated and primary care physicians were asked about possible diseases not recorded at baseline that occurred during the follow-up period.

**Setting:**

Center for Endocrinology, Diabetology, and Osteology at the University Hospital Marburg, Inpatient Department, Germany.

**Patients:**

All patients who presented to our center for the 72-hour fast between 2005 and 2018 and were discharged without an internal medicine diagnosis were included.

**Interventions:**

Survey by questionnaire, via telephone interview.

**Main outcome measures:**

Patient-reported information on current symptoms compared to original symptoms, diagnosis of insulinoma or diabetes mellitus during follow-up, matched with primary care physician data, and metabolic and biometric data such as body mass index (BMI), homeostasis model assessment for insulin resistance (HOMA IR), insulin sensitivity Matsuda Index (ISI-M), and area under the curve.

**Results:**

A total of 41 datasets were evaluated at baseline and 38 patients were followed for an average of approximately 10 years. In total, 61% of respondents still reported the same symptoms as at baseline. No insulinoma was missed in these patients. Only two of the 38 patients developed diabetes mellitus.

**Conclusion:**

The high percentage of patients who are discharged as “healthy” and still have symptoms after many years is disturbing. It is possible that the symptoms are not due to low blood glucose. We urge caution with use of the term “healthy”. We advocate a multidisciplinary therapeutic approach after an organic cause of hypoglycemia has been ruled out. Psychosomatic treatment seems to be useful. In addition, more research should be conducted on this topic.

## Introduction

Since the discovery of the islets of Langerhans in 1869, a veritable scientific avalanche of data and publications has been unleashed, significantly advancing our understanding of the endocrine and exocrine pancreas and its influence on carbohydrate metabolism. It was the culmination of this scientific effort that led to the development of insulin as a drug, which can now be purified, stabilized, and injected, thus making a decisive contribution to the treatment of people with diabetes mellitus [1, 2].

But how does this account square with spontaneous hypoglycemia, today also referred to as Whipple’s triad?

In 1924, Harris et al. described a complex of symptoms that included hunger, weakness, and nervous restlessness. Because they observed similar symptoms in patients receiving insulin, they concluded that it must be a disease associated with endogenous hyperinsulinemia [3]. Just 3 years later, Wilder et al. published a case of severe hypoglycemia caused by Langerhans cell carcinoma [4]. In a landmark manuscript published in 1935, Whipple et al. provided a comprehensive summary of the achievements in the field up to that time, focusing on insulinoma [5].

Shortly thereafter, the term Whipple’s triad was introduced and has served as a diagnostic compass ever since [6].

Although great medical successes have been achieved in the entity known as spontaneous hypoglycemia over the past few decades, it became clear after the publication of a comprehensive paper by Conn and Seltzer in 1955 that there might be some problems in its diagnosis and treatment [7].

The growing interest of the lay press, which has associated various and vague symptoms with questionable spontaneous hypoglycemia, has complicated the issue [8, 9]. In the late 1990s, Service et al. published a summary of the findings, controversies, and experience to date in the field of spontaneous hypoglycemia. They also proposed diagnostic pathways for the management of patients in different clinical settings [10, 11]. Since then, the 2009 Endocrine Society guideline has been viewed by many physicians and scientists as a beacon in the uncertain waters of spontaneous hypoglycemia [12].

We also implement these recommendations in our clinical practice [13].

At our center, we have extensive expertise in the diagnosis and treatment of patients with insulinomas [14, 15]. However, the patients we are seeing are many more than those who actually have an internal medicine diagnosis. We are hence concerned that we often cannot adequately help these patients who are discharged as “healthy” and, also, that this situation does not change over the years.

We believe it is time to turn the tide. In this paper, we take a look at those individuals who were discharged from our clinic as “healthy” and inquire how they are doing after many years.

## Materials and methods

### Patient selection

**Fig. 1:**
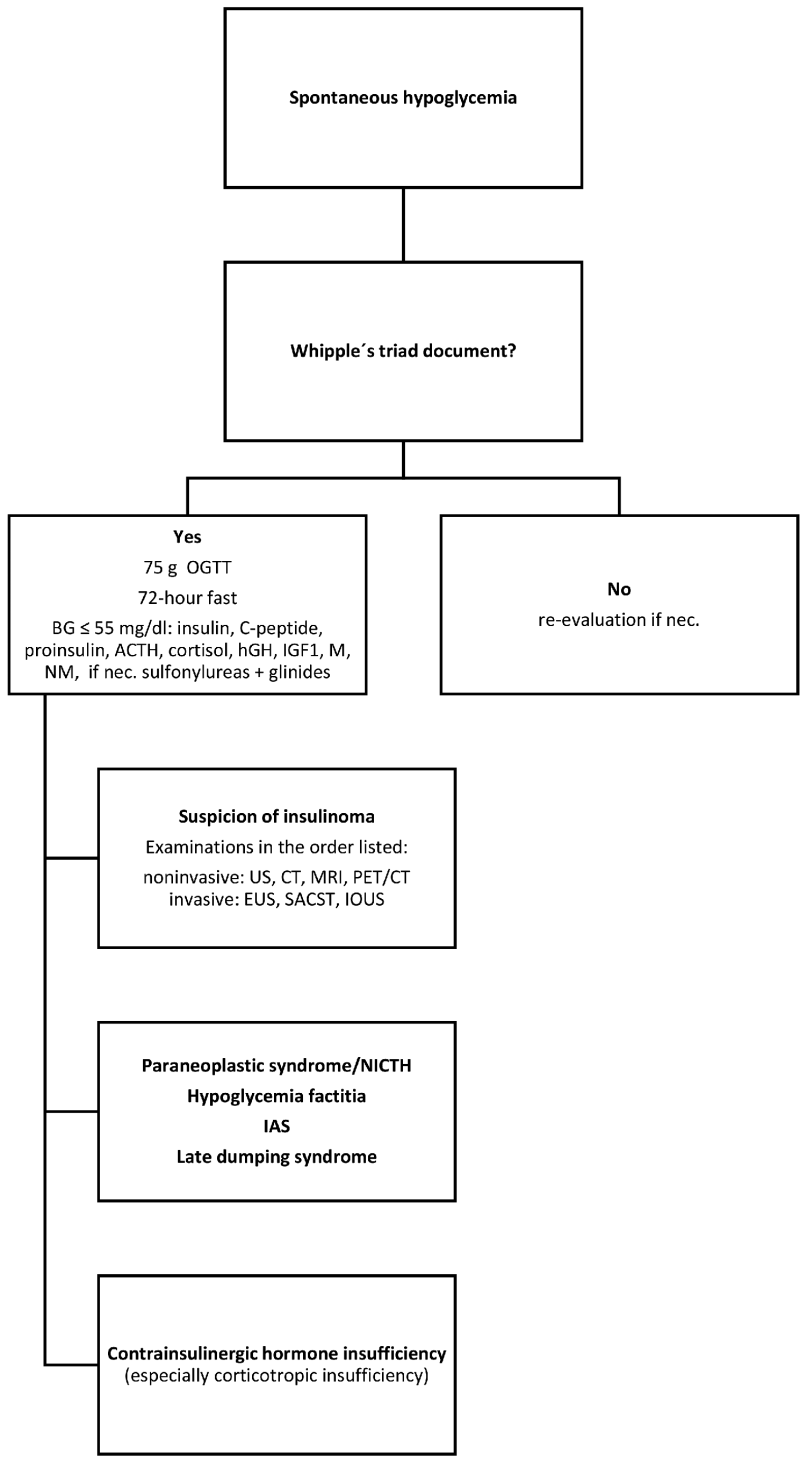
This flowchart represents a standardized sequence of our diagnostic program. The presence of Whipple´s triad is crucial for the initiation of further investigations. Based on the findings (hormonal constellations), various somatic diseases can be considered. *Abbreviations*: OGTT, oral glucose tolerance test; BG, blood glucose; ACTH, adrenocorticotropic hormone; hGH, human growth hormone; IGF-1, insulin-like growth factor 1; M, metanephrine; NM, normetanephrine; if nec., if necessary; US, ultrasonography; CT computer tomography; MRI, magnet resonance imaging; PET/CT, positron emissions tomography/ computer tomography; EUS, endoscopic ultrasonography; SACST, selective arterial calcium stimulation; IOUS, intraoperative ultrasonography; NICTH, non-islet cell tumor hypoglycemia; IAS, insulin autoimmune syndrome

As a first step, all patients were identified who presented to the Center for Endocrinology, Diabetology and Osteology at the University Hospital Marburg, Germany, between 2005 and 2018 for clarification of presumed hypoglycemia. A previously documented Whipple’s triad (blood glucose ≤ 55 mg/dl, neuroglycopenic symptoms, convalescence after blood glucose elevation) was required for inpatient diagnostic testing [16]. In our clinic, this diagnosis follows a standardized program (Fig. 1).

Data from these patients were then reviewed for the following inclusion criteria:

- Age of majority at time of data collection.
- No evidence of:
  - Insulinoma or other form of endogenous hyperinsulinism.
  - Dumping syndrome.
  - Pituitary or adrenal insufficiency.
  - Diabetes mellitus.

### Blood sampling, assays, and analysis

As part of the routine evaluation for spontaneous hypoglycemia, each patient received a prolonged 75 g oral glucose tolerance test (OGTT; 75 g dextrose monohydrate in 300 ml water). This included the determination of insulin and glucose at time points 0, 30, 60, 90, 120, 150, 180, 210, 240, 270, and 300 min, which were used for subsequent analyses. Insulin was tested with the CLIA-Test (chemiluminescence immunoassay, Elecsys Insulin, Roche Diagnostics GmbH, Mannheim, Germany). Blood glucose was measured using a Nova StatStrip® glucose meter. These data were used to calculate homeostasis model assessment for insulin resistance (HOMA IR) [17], insulin sensitivity Matsuda Index (ISI-M) [18], and area under the curve (AUC 0-300 min) of insulin using Simpsons rule to evaluate insulin secretion and resistance.

Body mass index (BMI) was determined by dividing the measured weight (kg) by the square of the measured height (m^2^).

Statistical analysis was performed with Microsoft Excel for Mac (version 16.29).

### Grouping and retrospective analysis

A retrospective analysis of demographic and medical data documented in the ORBIS® patient information system was performed. Patients were classified into two groups (NGT normal glucose tolerance and IGT impaired glucose tolerance) based on their glucose tolerance at that time. This classification was made according to the current guidelines [19].

Within these two groups, a further subdivision was made based on whether hypoglycemia.

(H = BG ≤ 55 mg/dl) was documented in the OGTT. This resulted in four groups (NGT H+; NGT H-; IGT H+; and IGT H-).

### Questionnaire and contact

We created a short and concise questionnaire that contained three sections, each with two to four questions. The purpose of this questionnaire was to find out if the complaints that prompted the patients’ presentation to our center were still present. In addition, biometric data were requested. Furthermore, it was asked whether an insulinoma or diabetes mellitus had been diagnosed in the meantime.

Subjects were first contacted by telephone and informed about the study. If they were interested in participating, the questionnaire and information, including an informed consent form, were sent by mail.

### Primary care physician data

For the purpose of quality assurance, the patients were requested to provide release of confidential information from their primary care physician. If this was available, the colleagues were contacted. The physicians were again explicitly asked about the presence of diabetes mellitus, including the current medication list or the diagnosis of an insulinoma.

## Results

### Cohort description

**Fig. 2:**
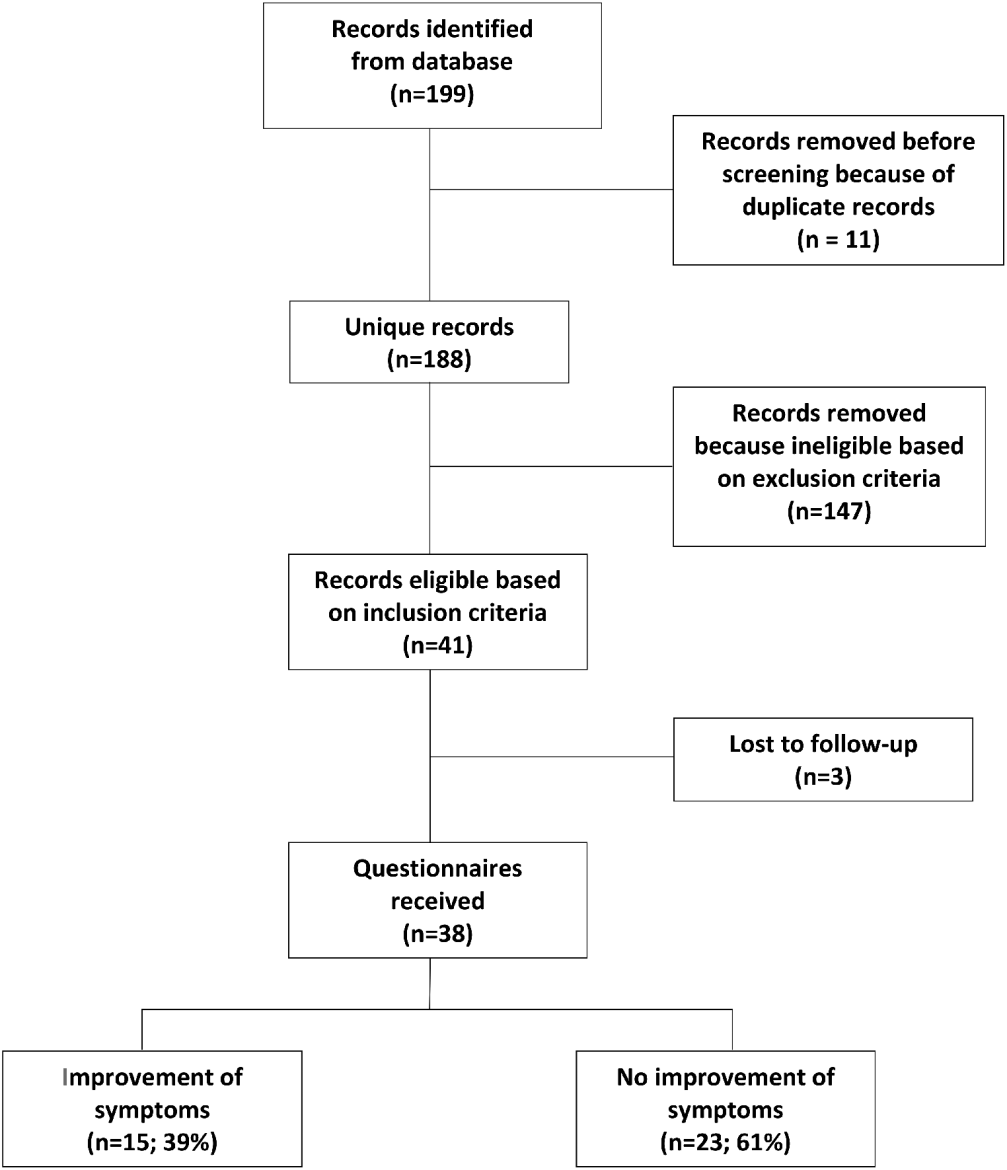
This graph shows how the datasets were handled and how the subjects eligible for the study were selected and grouped. It also shows that 38 patients eventually returned the questionnaire. Of these, 61% reported no improvement in symptoms

Between 2005 and 2018, 199 records of patients presenting to our center for investigation of spontaneous hypoglycemia were identified. Eleven were screened out due to multiple presentations, leaving 188. The inclusion criteria were met by 41 patients (22%). Of these, 76% were female. Three of them were subsequently lost to follow-up (Fig. 2).

The patients’ average age at the time of the study was 45 years. With a mean BMI of 24.46 kg/m^2^, weight was classified as normal [20].

### Group description and metabolic characteristics at time of consultation

Based on the above criteria, four groups were formed (Table 1).

**Table 1:** Comparison of data collected from study participants at the first medical examination (*t0*) and from the current data collection (*t1*)

	All participants		NGT H+		NGT H-		IGT H+		IGT H-	
	*n* = 41 m = 10 f = 31	*n* = 38 m = 10 f = 28	*n* = 7 m = 0 f = 7	*n* = 7 m = 0 f = 7	*n* = 10 m = 3 f = 7	*n* = 8 m = 3 f = 5	*n* = 6 m = 0 f = 6	*n* = 6 m = 0 f = 6	*n* = 18 m = 7 f = 11	*n* = 17 m = 7 f = 10
	t0	t1	t0	t1	t0	t1	t0	t1	t0	t1
Age (mean)	45 y	54 y	52 y	64 y	41 y	52 y	44 y	52 y	44 y	52 y
BMI (mean)	24.46	25.07	23.21	23.60	23.49	25.35	22.67	23.48	25.98	26.1
HOMA-IR (median)	1.51		0.74		1.52		1.05		2.00	
ISI-M (median)	5.82		8.81		7.82		5.56		4.47	
AUC insulin 0-300 min (mU/l) (median)	10,822		9767		9491		14,121		12,595	

*Abbreviations* AUC, area under the curve; BMI, body mass index; HOMA, homeostasis model assessment for insulin resistance; ISI-M insulin sensitivity Matsuda Index, t0, time of first medical examination; t1, time of data collection

The NGT H + group consisted entirely of women. On average, they were of normal weight and had low insulin resistance or good sensitivity.

Comparative figures apply to the NGT H- group. Here, 70% were female. They were also of normal weight and did not have insulin resistance.

The third group, IGT H+, was also 100% female. They had on average the lowest BMI of all subjects included, but the ISI-M was slightly lower and in the range of reduced insulin sensitivity.

The last group (IGT H-) had the lowest proportion of women (61%). Subjects in this group were slightly overweight on average and had the worst insulin resistance and sensitivity scores.

### Questionnaire and long-term follow-up

The mean follow-up was 9.58 years. Average BMI increased in this time by 0.61 (Table 1).

Only two patients reported being diagnosed with diabetes mellitus during the study. These patients were in the NGT H- and IGT H + groups.

In total, 61% of respondents reported that the symptoms that led to the diagnosis were still present.

Respondents were also asked which of these patients had received a treatment recommendation. However, a more detailed analysis was not possible. Of the 23 patients who did not experience any improvement in their symptoms, only about half (52%) received a treatment recommendation. On the one hand, these recommendations include dietary therapy, such as eating several small meals with a reduction in rapidly absorbed carbohydrates (e.g., avoiding sweet drinks and wheat flour products). On the other hand, off-label therapy with metformin to break insulin resistance and acarbose had been recommended only in rare cases.

Of those who reported symptom improvement, 60% were prescribed therapy. For the remainder, symptoms resolved without further intervention.

## Discussion

For more than 100 years, diagnosis of spontaneous hypoglycemia has been a major challenge for physicians around the world [21]. In particular, the wide range of symptoms and complaints and the diverse constellations of laboratory findings and clinical conditions mean that correct diagnosis and subsequent treatment can sometimes take a very long time [22]. There are many “atypical” case reports documenting the difficulties in making a correct diagnosis [23–25].

Recently, one of our patients (who was not included in the study cohort) was treated for 6 months for undefinable epilepsy due to recurrent unexplained loss of consciousness.

An attempt was made to explain the epilepsy in the context of autoimmune encephalitis, but this could not be confirmed due to negative antibodies. We first met this patient in our emergency room. She was admitted for a new focal epileptic seizure. At the same time, the patient was found to have a blood glucose level of 36 mg/dl. It took 1.5 h for symptomatic hypoglycemia to develop. An insulinoma was then discovered in the pancreatic head and surgically removed. The symptoms subsequently resolved.

Stories like this one in which patients have gone through an odyssey of doctor visits abound in the literature [26]. . For the physician who deals with these problems, such vicissitudes are like a treasure hunt.

However, special attention should be paid to those who were discharged as “healthy”. It is for this reason that, in the present study, only patients in whom no organic cause could be found for the reported complaints were included. Before we started this work, we were concerned that these patients would fall through the cracks. For example, a diagnosis of insulinoma usually leads to surgery, hypopituitarism to endocrinological care, and so on. In addition, those who were diagnosed with diabetes mellitus during the diagnostic process were excluded. However, this was not primarily due to latent diabetes being proposed as an explanation for hypoglycemia, as this has long been known [27, 28]. Rather, we believe that a diagnosis of diabetes mellitus usually results in patients remaining under medical care [19].

That this is not only a problem in our center can be deduced from several publications. In one paper, Wiesli et al. examined the 72-hour fasts of 23 patients who presented to their center for evaluation of spontaneous hypoglycemia [29]. The focus of the latter manuscript was, of course, on the seven patients who could be diagnosed with an insulinoma. The rest were referred to as individuals with “normal” health. Similarly, Quinkler et al. analyzed their data dating back to between 1970 and 2004 and identified 39 patients with insulinoma during this period. However, 150 of their patients were described as “healthy” in this publication even though they had symptoms [30].

These numbers are reflected in our own data. A total of 45% of our patients were discharged without a clear diagnosis.

Given this significant number, the question arises as to how this discrepancy between the number of those who initially present for testing and those who cannot be diagnosed transpires.

On the one hand, one might deduce that the tests “missed” something.

Perhaps the 72-hour fast is not sensitive enough and insulinomas are missed despite all the care taken [31]. This may be due to pulsatile insulin secretion [32]. A critical cell mass that causes symptoms when it is above a certain size has also been discussed in the past [33]. Proper diagnosis of contrainsulinergic hormone imbalances can also be problematic [13]. Possibly the tests were not sensitive enough to identify proinsulinomas [34, 35]?

However, in the patient cohort studied herein, no evidence was found that any of these entities were diagnosed during the course of the disease, although not all patients could be reached.

As this information may be subject to error, the general practitioners were also contacted for the purpose of quality assurance. Again, no one reported that an organic cause was identified during the course of the study.

These findings are consistent with the limited data available. Van Bon et al. were able to follow 76% of their patients, in whom no insulinoma was initially found, for a period of 53 months. No insulinoma occurred during this period [36]. Wiesli et al. documented the fact that none of the 16 patients who had no neuroglucopenic symptoms during the 72-hour fast required emergency treatment for hypoglycemia in the following 19 months [29].

Looking at these data in isolation one might infer that this is good news.

Obviously, no major pathology was missed, and the sensitivity of the 72-hour fast is close to the reported 100% [37]. Since the 72-hour fast is the gold standard for the diagnosis of endogenous hyperinsulinemia, no external criterion can be used for the test.

Given that no external criterion can be used to verify the test, the validity of the test can be verified only via long-term observation [38].

Although no one in the study population was found to have a “missed” organic disease, a certain proportion of patients still suffer from the same complaints.

Several aspects should be considered in order to find an explanatory approach to resolve this conundrum.

In the group of patients we studied, 13 patients had hypoglycemia on OGTT, which we diagnosed as reactive hypoglycemia. All were female and of normal weight. Similar results were reported by Wiesli et al. [21], Altuntas et al. [25], and, already in 1975, by Hofeldt et al. [8]. This is probably due in part to the diagnostic imprecision of the OGTT. In a study by Berlin et al., hypoglycemia was induced in 10% of healthy subjects [39]. As a result, OGTT has been heavily criticized and is no longer recommended for diagnostic purposes [40–42]. The mixed meal tolerance test (MMTT) is generally considered to be a valid diagnostic tool [12, 43]. However, diagnostic difficulties may arise. For example, its sensitivity was questioned by Hogan et al. after failure to provoke hypoglycemia in 33 patients with a suspected diagnosis of reactive hypoglycemia [44]. Another problem is the lack of standardization of MMTT as there is no clear recommendation for meal composition [42].

Although use of the OGTT for diagnostic purposes has been criticized, it is a proven tool for determining insulin resistance and insulin secretion [45–47].

The data analyzed showed that individuals with normal glucose tolerance who were hypoglycemic on the OGTT had the least insulin resistance in comparison. The patients with impaired glucose tolerance, who had both lower insulin resistance and lower blood glucose levels, showed increased insulin secretion, this possibly pointing to better beta cell function. Furthermore, these calculations were not used at the time and were prepared only as part of the current study.

Retrospectively, 70% of the hypoglycemic patients received a treatment recommendation for postprandial syndrome [48]. However, less than half of these patients reported an improvement in symptoms as a result of the treatment recommendation.

In total, 48% of the remaining 25 patients who did not have a hypoglycemic OGTT also received a nutritional therapy recommendation. This resulted in symptom improvement in 41%.

In our study population, it was notable that only one patient each in the NGT and IGT groups (5%) developed diabetes mellitus. This is not consistent with the data of Abdul-Ghani et al. who observed the development of diabetes mellitus in approximately 12% of patients with IGT over a period of 7–8 years [49]. This difference could be explained by the dietary recommendations or the recommendation for drug therapy with metformin among our patients.

Regarding symptoms, Ford et al. in 1976 suggested that patients with hypoglycemic complaints for which no organic cause could be found may suffer from psychiatric disorders [50]. Johnson et al. also faced this problem and postulated that there is likely a high proportion of psychiatric disorders in these patients and that the symptoms overlap [9]. In addition, psychosomatic or psychiatric disorders are mentioned as differential diagnoses by some authors [51, 52].

In summary, the data suggest that the 72-hour fasting test does not miss anyone with an organic cause of hypoglycemia. On the other hand, it is possible that there are patients who are sensitive to a higher glucose load, but this cannot or should not be considered pathological. There is also the possibility that a certain number of people in this group of patients may have a psychosomatic or psychiatric disorder that mimics or exacerbates neuroglycopenic symptoms [53].

These patients have symptoms that can be interpreted as hypoglycemic complaints but are not ultimately due to low blood glucose. Of course, quite possibly, patient general information leads to many patients presenting with diffuse complaints [54].

This assumption was confirmed as early as in 1973 in a statement by the American Diabetes Association, the Endocrine Society, and the American Medical Association [47].

Obviously, this is a problem that has been known for decades.

Thus, what could have been done to improve the care of patients discharged as healthy given that some still have complaints?

Farahmand et al. recommended better screening with a special questionnaire before initiating a 72-hour fast. They cited the high overhead costs of “unnecessary” 72-hour fasts as the main incentive for improvement [55]. However, this questionnaire does not include items related to possible psychosomatic/psychiatric symptoms.

Screening for psychiatric disorders prior to evaluation resulting in these patients not receiving an internal medicine evaluation would, in our opinion, be negligent.

We agree with Griffiths et al., who recommend that all patients with hypoglycemic and neuroglycopenic complaints should be evaluated [56].

Finally, in our opinion, it is not acceptable to exclude certain patients in advance. Rather, a careful internal examination should be performed, after which the potential for a different etiology of the complaints may be considered. This should be openly discussed with the patient, while, furthermore, psychosomatic/psychiatric evaluation should be recommended. Interdisciplinary collaboration with colleagues in appropriate specialties is essential.

The term “healthy” should be used with caution and only after a guideline-based medical evaluation that includes both an oral carbohydrate load and a 72-hour fast. In addition, other causes that may explain the symptoms should be included in the medical evaluation, such as a psychosomatic evaluation. Another option would be to require patients to return after a certain period of time for a basic check-up. It is of importance for the patient to have a good relationship with his/her primary care physician or an established specialist who would see him/her on an outpatient basis.

Of course, we can only speak for our own center. Perhaps there are departments that are already following this path. In addition, we deal with a limited number of patients. The inclusion criteria are also freely chosen and the thoughts that led to their creation should be discussed.

Nevertheless, we believe that there should be further interdisciplinary research in order to be able to help patients in the best possible way in the future.

## Data Availability

The data that support the results (findings) of this study are available from the corresponding author upon reasonable request.
